# Bipolar Disorder and Parkinson disease: a 123I-FP-CIT SPECT study

**DOI:** 10.1192/j.eurpsy.2023.1090

**Published:** 2023-07-19

**Authors:** G. D’agostino, G. Cascino, A. M. Landolfi, R. Erro, P. Barone, P. Monteleone

**Affiliations:** 1 Medicine, Surgery and Dentistry, “Scuola Medica Salernitana”, University of Salerno, Baronissi; 2 Medicine, Surgery and Dentistry, “Scuola Medica Salernitana”, University of Salerno, Baronissi (SA); 3 Medicine,Surgery and Dentistry, “Scuola Medica Salernitana”, University of Salerno, Baronissi, Italy

## Abstract

**Introduction:**

Bipolar Disorder (BD) has been suggested to be a risk factor for development of Parkinson Disease. Psychiatric drugs used as standard treatment of BD includes many drugs that are known to induce drug-induced parkinsonism (DIP).

**Objectives:**

Clinical differentiation between PD and DIP is a clinical and scientific crucial result. It might be aided by functional neuroimaging of the dopaminergic nigrostriatal pathway.

**Methods:**

Twenty consecutive BD patients with parkinsonism were clinically assessed and underwent ^123^I-ioflupane dopamine transporter SPECT. Imaging data of BD patients with pathological nigrostriatal pathway were further compared to a population of *de-novo* PD patients.

**Results:**

Four BD patients had abnormal scans; they had higher putaminal binding ratio and putamen-to-caudate ratios than PD patients, despite similar motor symptom burden.

**Conclusions:**

in our initial results, up to 20% of BD patients with parkinsonism might have an underlying dopaminergic deficit, which is higher than excepted in the general population. This evidences supports that BD represents a risk factor for subsequent development of neurodegenerative parkinsonism.

**Disclosure of Interest:**

None Declared

